# Mitochondrial dynamics: updates and perspectives

**DOI:** 10.1038/s41598-024-59998-1

**Published:** 2024-04-30

**Authors:** Kezhong Zhang, Ježek Petr

**Affiliations:** 1 Center for Molecular Medicine and Genetics, Wayne State University School of Medicine, Detroit, MI 48201 USA; 2 Department of Biochemistry, Microbiology, and Immunology, Wayne State University School of Medicine, Detroit, MI 48201 USA; 3 Laboratory of Mitochondrial Physiology, Institute of Physiology of the Czech Academy of Sciences, Vídeňská 1083, 14220 Prague, Czech Republic

**Keywords:** Mitochondria, Molecular medicine

## Abstract

Mitochondria, the powerhouse and the vital signaling hub of the cell, participate in a variety of biological processes, such as apoptosis, redox responses, cell senescence, autophagy, and iron homeostasis. Mitochondria form a mostly tubular network, made up of an outer and a cristeae-forming inner membrane. The network undergoes dynamic fusion and fission that change its morphological structure according to the functional needs. Approximately 1500 mitochondrial proteins encoded by nuclear genome plus over 10 proteins encoded by mitochondrial DNA are folded and assembled in the mitochondria under a high-fidelity control system. These proteins are involved in oxidative phosphorylation, metabolism, network and cristae dynamics, mitophagy, import machinery, ion channels, and mitochondrial DNA maintenance. This Collection gathers original research that advances our understanding of the monitoring techniques and pathophysiological significance of mitochondrial dynamics in health and disease.

Understanding the regulation and functional impact of mitochondrial dynamics has important implications in the understanding and treatment of human complex diseases, in which mitochondria act as a major player. In this direction, we have put together a collection of timely research articles with a focus on the monitoring technique, regulation, and pathophysiological impact of mitochondrial dynamics.

In this collection, Smith et al.^1^ described their efforts on tickling the technical difficulty in detecting dynamic ultrastructural changes of mitochondria in live cells. They utilized fluorescence lifetime imaging and anisotropy spectroscopy of mitochondrial NADH to investigate mitochondrial cristae alignment. This technology enables measuring highly ordered and directional NADH patterns to indicate electron and photonic cascade, communication, and dynamic structural changes of mitochondria. In regard to the association of mitochondrial DNA copy number changes with nuclear and mitochondrial genetic variants, Koller et al.^2^ identified two genome-wide significant autosomal loci associated with qPCR-measured mitochondrial DNA copy numbers at the *HBS1L* and *GSDMA* genes. They found that the mitochondrial genome itself contributed only marginally to mtDNA-CN regulation and that mitochondrial haplogroups do not exert any significant influence on mtDNA-CN.

Cellular senescence is a therapeutic endpoint in melanoma, and the senescence-associated secretory phenotype can affect tumor growth and microenvironment. In this collection, Tarallo et al.^3^ demonstrated that silencing of the mitochondria fusion protein mitofusin 1 (MFN1) decreases the senescent-associated secretory phenotype, promotes immune cell recruitment, and delays melanoma tumor growth after chemotherapy.

In terms of pathophysiological relevance of mitochondrial DNA dynamics, Lai et al.^4^ showed that mitochondria-localized ubiquitin-specific protease 18 (USP18) enhances Dengue virus (DENV) replication by regulating the release of mitochondrial DNA (mtDNA) to promote viral replication. The mechanisms by which USP18 stimulates DENV replication include enhanced mitochondrial reactive oxygen species (ROS) production, changes in mitochondrial membrane potential, mtDNA oxidation and fragmentation, as well as increased mitochondrial permeability. Additionally, DeFoor et al.^5^ investigated the effect of Remdesivir, an FDA-approved drug to treat COVID-19, on mtDNA-CN changes. Interestingly, Remdesivir treatment increased mtDNA-CN in Mv1Lu cells or rodent livers but did not significantly affect mitochondrial function.

Mitochondrial DNA copy number (mtDNA-CN) decline is a hallmark of aging, which is associated with the development of Alzheimer’s disease (AD)^6,7^. In this collection, Lynch et al.^8^ identified strong associations between mtDNA-CN and clinical measures of AD. Particularly, the alteration of mtDNA-CN was strongly associated with global AD pathology and tau tangles.

To summarize, we anticipate that this collection of research articles from the front-running scientists will help the research community get a glimpse of the advances in the pathophysiological relevance of mitochondrial dynamics. While much work in understanding the regulation and functional significance of mitochondrial dynamics remains to be done, we hope that this collection will trigger more interest in this important research topic.

We thank all contributors of this Collection for their works.

## References

[1] Smith HE (2024). The use of NADH anisotropy to investigate mitochondrial cristae alignment. Sci. Rep..

[2] Koller A (2024). Nuclear and mitochondrial genetic variants associated with mitochondrial DNA copy number. Sci. Rep..

[3] Tarallo D (2024). Mitofusin 1 silencing decreases the senescent associated secretory phenotype, promotes immune cell recruitment and delays melanoma tumor growth after chemotherapy. Sci. Rep..

[4] Lai J-H (2023). USP18 enhances dengue virus replication by regulating mitochondrial DNA release. Sci. Rep..

[5] DeFoor N (2023). Remdesivir increases mtDNA copy number causing mild alterations to oxidative phosphorylation. Sci. Rep..

[6] Lopez-Otin C, Blasco MA, Partridge L, Serrano M, Kroemer G (2023). Hallmarks of aging: an expanding universe. Cell.

[7] Gao R, Ma SL (2022). Is mitochondria DNA variation a biomarker for AD?. Genes (Basel).

[8] Lynch MT (2023). Evaluating genomic signatures of aging in brain tissue as it relates to Alzheimer’s disease. Sci. Rep..

[9] Taylor SW (2003). Characterization of the human heart mitochondrial proteome. Nat. Biotechnol..

